# Muscle Oxidative Stress Plays a Role in Hyperthyroidism-Linked Insulin Resistance

**DOI:** 10.3390/antiox12030592

**Published:** 2023-02-27

**Authors:** Gianluca Fasciolo, Gaetana Napolitano, Marianna Aprile, Simona Cataldi, Valerio Costa, Maria Teresa Muscari Tomajoli, Assunta Lombardi, Sergio Di Meo, Paola Venditti

**Affiliations:** 1 Dipartimento di Biologia, Università di Napoli Federico II, 80126 Naples, Italy; 2 Dipartimento di Scienze e Tecnologie, Centro Direzionale, Università degli Studi di Napoli Parthenope, Isola C4, 80143 Naples, Italy; 3 International PhD Programme, UNESCO Chair “Environment, Resources and Sustainable Development”, Department of Science and Technology, Parthenope University of Naples, 80143 Naples, Italy; 4 Institute of Genetics and Biophysics “Adriano Buzzati Traverso”, National Research Council, Pietro Castellino Street 111, 80131 Naples, Italy

**Keywords:** vitamin E, AKT, JNK, NRF1, PGC1-α, BIP, EIF, *Slc2a1*, *Slc2a4*, *Ppara*, *Pparg*, *Cd36*, *Il1b*

## Abstract

While a low level of ROS plays a role in cellular regulatory processes, a high level can lead to oxidative stress and cellular dysfunction. Insulin resistance (IR) is one of the dysfunctions in which oxidative stress occurs and, until now, the factors underlying the correlation between oxidative stress and IR were unclear and incomplete. This study aims to explore this correlation in skeletal muscle, a tissue relevant to insulin-mediated glucose disposal, using the hyperthyroid rat as a model of oxidative stress. The development of IR in the liver from hyperthyroid animals has been widely reported, whereas data concerning the muscle are quite controversial. Thus, we investigated whether hyperthyroidism induces IR in skeletal muscle and the role of oxidative stress in this process. Particularly, we compared the effects of hyperthyroidism on IR both in the absence and presence of vitamin E (Vit E), acting as an antioxidant. Putative correlations between ROS production, oxidative stress markers, antioxidant capacity and changes in intracellular signalling pathways related to insulin action (AKT) and cellular stress response (EIF2α; JNK; PGC1α; BIP; and NRF1) were investigated. Moreover, we assessed the effects of hyperthyroidism and Vit E on the expression levels of genes encoding for glucose transporters (*Slc2a1*; *Slc2a4*), factors involved in lipid homeostasis and insulin signalling (*Pparg; Ppara, Cd36*), as well as for one of the IR-related inflammatory factors, i.e., interleukin 1b (*Il1b*). Our results suggest that hyperthyroidism-linked oxidative stress plays a role in IR development in muscle and that an adequate antioxidant status, obtained by vitamin E supplementation, that mitigates oxidative stress, may prevent IR development.

## 1. Introduction

Reactive oxygen species (ROS) represent by-products of aerobic metabolism and, at low concentrations, are involved in several regulatory processes [1]. However, due to their reactive nature, which makes them capable of oxidizing and damaging biomolecules, they can lead to cellular and tissue dysfunction [2]. Therefore, the cellular ROS content is finely regulated, and the evolution of aerobic life has gone hand in hand with that of an antioxidant defense system to protect cells from the action of ROS [1]. The cellular antioxidant defense system comprising enzymatic and non-enzymatic systems contributes to establishing a dynamic balance between ROS production and removal (redox homeostasis) [1].

In various physio-pathological states, an imbalance between ROS production and their removal can cause oxidatively damaged macromolecule accumulation, which predisposes to cellular and tissue dysfunction [2].

Insulin resistance (IR) is one of the dysfunctions in which oxidative stress occurs [3]. In skeletal muscle and adipocytes, the translocation of glucose transporter 4 (GLUT4) from the cytoplasm to the plasma membrane is triggered by insulin and requires the activation of signaling pathways, involving the phosphatidyl inositol 3 kinases (PI3K) and protein kinase B (PKB, also known as AKT) [4].

In insulin-resistant cells, the impaired insulin response leads to a reduced GLUT4 translocation on the plasma membrane with a consequent increase in blood glucose levels. In turn, the pancreas secretes higher amounts of insulin, activating a positive feedback loop which results in high plasma insulin levels and insulin desensitization of peripheral tissues [4].

IR can be the result of various systemic insults, many of which determine increased oxidative stress, including increased caloric intake [5], sedentary lifestyle [6] and inflammation [7].

Although the link between oxidative stress and IR is of great interest, the knowledge about factors underlying this correlation is still unclear or incomplete, as well as the role played by oxidative stress as a cause or a consequence of IR.

Given the pivotal role of skeletal muscle in insulin-mediated glucose disposal, our study aims to assess in this tissue the correlation between IR and oxidative stress. To this aim, we used the hyperthyroid rat as an oxidative stress model. Thyroid hormones contribute to glucose homeostasis, but, in the hyperthyroid state, a well-known condition called thyroid diabetes [8] can develop. Thyroid diabetes could conceivably be a consequence of thyroid-hormone-induced oxidative stress [4]. In hyperthyroid animals, the development of IR in the liver tissue has been widely reported [4], while data on muscles are quite controversial, reporting both increases [9,10,11] and reductions [12] in glucose absorption.

Specifically, we studied whether hyperthyroidism could induce IR in the skeletal muscle and the contribution of the oxidative stress to this process. For this purpose, we directly compared two conditions in which the extent of oxidative stress is different, i.e., absence or presence of antioxidant supplementation with Vit E, which reinforces the antioxidant defense and attenuates the oxidative stress.

Notably, we verified the existence of correlations among ROS production, markers of oxidative stress, antioxidant capacities and changes in intracellular signaling pathways related to insulin action (phosphorylation of serine/threonine kinase AKT), and the cellular stress response (Eukaryotic Translation Initiation Factor 2α, EIF2α; c-jun NH_2_ terminal kinases, JNK; Peroxisome proliferator-activated receptor-Gamma Coactivator 1α, PGC-1α; Binding Immunoglobulin Protein, BIP; and Nuclear Respiratory Factor 1, NRF1). Furthermore, we evaluated the effects of hyperthyroidism and Vit E on the expression levels of genes encoding glucose transporters (Solute carrier family 2 member 1, *Slc2a1*, encoding Glut1; Solute carrier family 2 member 4, *Slc2a4*, encoding Glut4), factors involved in lipid homeostasis (*five*), as well as for the interleukin 1b (*Il1b*) strongly correlated with IR [13,14,15].

Overall, our results suggest that oxidative stress contributes to the development of muscle IR since the attenuation of oxidative stress due to vitamin E supplementation can prevent the development of IR.

## 2. Materials and Methods

### 2.1. Animals

The “Ethical-Scientific Committee for Animal Experimentation” of the University of Naples Federico II and the Italian Minister of Health approved all experimental procedures on animals (authorization n° 836/2019 PR). Thirty-two male Wistar rats (Envigo, Bresso, Italy) were used for the experiments and fed a control diet (Mucedola, Milan, Italy), which contained 70 mg/kg of α-tocopherol until day 80 of age. They were then divided into four groups: rats fed the control diet for ten days (C); rats fed a diet supplemented with 700 mg/kg of α-tocopherol (C + VE) for ten days; and rats rendered hyperthyroid (H) by intraperitoneal administration of triiodothyronine (T3, Sigma-Aldrich, St. Louis, MO, USA), 50 μg/100 body weight) for ten days; rats made hyperthyroid and fed the α-tocopherol (H + VE) enriched diet. Thyroid hormone treatment was chosen considering that IR occurs after ten days of such a T_3_ administration [16]. The Vit E treatment was chosen because it attenuates both oxidative damage [17] and, also thyroid-hormone-induced alterations of rat heart electrical activity and contraction frequency [18]. Rats were housed at 24 ± 1 °C, with a constant artificial circadian cycle of 12 h of light and 12 h of dark, and 55 ± 10% relative humidity, and received water and food ad libitum. At the end of the experimental period, the glucose tolerance test was performed (as described below) and, the following day, the animals were euthanized after sedation with an intraperitoneal injection of Zoletil (60 mg/kg of body weight). Gastrocnemius muscles were harvested from both hind legs and placed in a beaker containing homogenization solution (HM) (220 mM mannitol, 70 mM sucrose, 1 mM EDTA, 0.1% fatty acid-free albumin, 10 mM Tris, pH 7.4) placed on ice.

### 2.2. Glucose Tolerance Test

Venus blood samples were obtained after 6 h of fasting from a small cut on the tail to determine basal glycemia and insulinemia. Then, after administering a glucose load (2 g/kg bw, i.p. injection), blood was drawn after 15, 30, 60, 90 and 120 min. All samples were centrifuged at 1400× *g* for 8 min at 4 °C and separated plasma was stored at −20 °C. Glucose and insulin levels were determined by a glucometer and a commercial ELISA kit (Mercodia, Winston Salem, NC, USA), rat insulin), respectively.

### 2.3. Tissue Preparations

Gastrocnemius muscles were cleaned from adipose and connective tissue, weighed, minced, and washed with HM. The tissue fragments were incubated with HM containing 0.1 mg/mL Protease (Sigma, St. Louis, MO, USA)) for 5 min. Then, they were rinsed and gently homogenized in HM (1:10 *w*/*v*) with a Potter Elvejem homogenizer at a speed of 500 rpm, for 2 min. The Vit E content was determined in aliquots of homogenate deproteinized with methanol and subjected to extraction with n-hexane. The hexane was evaporated in the presence of N_2_ at 40 °C, and the residues were resuspended in ethanol. The Lang [19] HPLC procedure was used to determine the Vit E content, and quantification was achieved using an external standard.

Other aliquots of the muscle homogenates were used either for the other assays or to isolate the mitochondrial fractions.

### 2.4. Isolation of Muscle Mitochondria

The supernatant obtained using centrifugation of the muscle homogenates (500× *g* for 10 min at 4 °C) to remove cell debris and nuclei was centrifuged at 3000× *g* for 10 min at 4 °C to obtain the mitochondrial pellets. Mitochondria were washed twice in washing buffer (WB) (220 mM mannitol, 70 mM sucrose, 1 mM EGTA, 20 mM Tris, pH 7.4) and finally resuspended in WB. The protein content in the mitochondrial fraction was determined with the biuret method.

### 2.5. Oxidative Damage Assessment and In Vitro Susceptibility to Oxidative Stress

Lipid and protein oxidative damages were determined in both muscle homogenates and mitochondria by determining the lipid hydroperoxides (Hps) levels [20], and protein-bound carbonyls (CO) [21,22].

After exposure of homogenates and mitochondria to Fe and ascorbate (Fe/Asc, 100/1000 μM, respectively) for 10 min, the susceptibility to oxidative stress was assessed by measuring the changes in hydroperoxides content [22].

### 2.6. Total ROS Content

The total ROS level was assessed by measuring the ROS-induced conversion of the non-fluorescent 2′,7′-dichlorodihydrofluorescin diacetate (DCFH-DA) in the fluorescent dichlorofluorescein (DCF), according to Driver [23] and as we previously described [22]. Briefly, 12.5 µg of homogenate proteins was incubated for 15 min in 10 µM of DCFH-DA in monobasic phosphate buffer 0.1 M, pH 7.4. After the addition of 100 µM of FeCl_3,_ the mixture was incubated for 30 min. The production of the fluorescent DCF (485 excitation wavelength, 530 emission wavelength) was measured with a multimode microplate reader (Synergy™ HTX Multimode Microplate Reader, BioTek (Winooski, VT, USA).

### 2.7. NADPH Oxidase (NOX) Activity Assay

The activity of the NADPH oxidase was assessed in homogenate samples [24,25] by determining the reduction in ferricytochrome c acetylated (80 μM) at 550, at room temperature, with NADPH as a substrate. The activity of the enzyme was calculated as the difference between the values obtained in the presence and absence superoxide dismutase, 100 μg/mL.

### 2.8. H_2_O_2_ Mitochondrial Release

The rate of the release of H_2_O_2_ from mitochondria was assessed through measuring at 30 °C the increase in fluorescence due to H_2_O_2_-induced oxidation of p-hydroxyphenylacetate (PHPA, excitation at 320 nm, emission at 400 nm) catalyzed by horseradish peroxidase (HRP) [26] by a fluorometer (JASCO Deutschland GmbH, Pfungstadt, Germany). Mitochondrial proteins (0.1 mg ∙mL^−1^) were incubated in a buffer containing HRP 6 UmL, PHPA 200 μg/mL, KCl 145 mM, Hepes 30 mM, KH_2_PO_4_ 5 mM, MgCl_2_ 3 mM, EGTA 0.1 mM, 1% BSA, and pH 7.4. Mitochondrial H_2_O_2_ release was assessed on respiring mitochondria using as respiratory substrates 10 mM pyruvate plus 2.5 mM malate in the absence (basal respiration) or in the presence of 500 μM ADP (ADP-stimulated respiration).

### 2.9. Antioxidant Enzymes Activities

The activity of the glutathione peroxidase (GPX) was determined at 37 °C using H_2_O_2_ and reduced glutathione (GSH) as substrates and measuring the rate of NADPH oxidation catalysed by glutathione reductase (GR) which reduces the oxidized glutathione (GSSG) obtained by the reaction [27], using a multi-mode microplate reader (Synergy™ HTX Multi-Mode Microplate Reader, BioTek).

Similarly, glutathione reductase activity (GR) was measured at 37 °C using GSSG as a substrate and assessing the oxidation rate of NADPH [28] using the microplate reader. Catalase activity was assessed according to Aebi [29].

Superoxide dismutase specific activity was measured at 25 °C assessing the decrease in the reduction rate of cytochrome c at 550 nm due to the superoxide radicals, produced by the xanthine–xanthine oxidase system. In brief, muscle homogenates were added to a solution containing 0.1 mM EDTA, 2 mM KCN, 50 mM KH_2_PO_4_, pH 7.8, 20 mM cytochrome c, 5 mM xanthine, and 0.01 U of xanthine oxidase. A unit of SOD activity corresponds to the enzyme concentration able to inhibit the reduction in cytochrome c by 50% in the presence of xanthine + xanthine oxidase [30].

### 2.10. Tissue and Mitochondrial Respiration

Tissue and mitochondrial respiration were assessed at 30° by an Hansatech respirometer in 1.0 mL of incubation medium (145 mM KCl, 30 mM Hepes, 5 mM KH_2_PO_4_, 3 mM MgCl_2_, 0.1 mM EGTA, 1% BSA, pH 7.4) containing 50 μL of 20% (*w*/*v*) homogenate or 0.25 mg/mL of mitochondrial protein. Pyruvate plus malate (10 and 2.5 mM, respectively) were used as respiratory substrates, in absence (basal respiration) or in presence (ADP-stimulated respiration) of ADP (500 μM).

### 2.11. Response of Skeletal Muscle to Insulin and Immunoblotting Analyses

Information on muscle sensitivity to insulin were obtained by exposing pieces of gastrocnemius muscle (50 mg) to 1 μM insulin (Sigma-Aldrich) for 30 min at 37 °C in a Krebs solution according to Amouzou et al., 2016 [31]. At the end of incubation period, the muscle pieces were lysed.

Particularly, muscle fragments were incubated for 15 min in a lysis buffer (pH 8, containing 150 mM NaCl, 50 mM Tris-HCl, 0.5% nonidet P-40, 0.5% sodium deoxycholate, 0.1% SDS) supplemented with Tissue Protease Inhibitor Cocktail (Sigma-Aldrich, 1:500, *v*/*v*). Lysates were centrifuged at 12,000× *g* for 30 min at 4 °C, and protein concentration was assessed by the biuret method. Immunoblotting was performed as previously reported [32], using the following primary antibodies: p-AKT (sc-377556, Santa Cruz, San Diego, CA, USA), Akt (sc-81434, Santa Cruz, San Diego, CA, USA), JNK (sc-7345, Santa Cruz, San Diego, CA, USA), p-JNK(sc-6254, Santa Cruz, San Diego, CA, USA)); EIF2α (L57A5, Cell Signaling Technology, Danvers, MA, USA); EIF2αP (9722, Cell Signaling Technology); GRP78 BIP (C50B12, Cell Signaling Technology); PGC-1 (sc-13067, Santa Cruz, San Diego, CA, USA), NRF1 (sc-33771, Santa Cruz, San Diego, CA, USA), and β-actin (A2066, Sigma-Aldrich, St. Louis, MO, USA). Secondary antibodies were from Sigma- Aldrich (sc-2030, Santa Cruz, San Diego, CA, USA). The excellent chemiluminescent detection Kit (ElabScience, Microtech, Naples, Italy) was used for the analysis and visualization of the immunoreactive bands, according to the manufacturer’s instructions. Densitometry data were generated analyzing ChemiDoc images or digital images of X-ray films exposed to immunostained membranes, and signal quantification was performed by Un-Scan-It gel software (Silk Scientific, UT, USA). The protein expression levels, a standard control sample was run on each gel, and all test values were compared to the expression levels analyzed in the control sample (control value = 1).

### 2.12. RNA Isolation, RT-PCR, and qPCR

“TRIzol Reagent” (Thermo Fisher Scientific, Waltham, Massachusetts, MN, USA) was used for total RNA isolation from skeletal muscle samples after tissue homogenization, according to the manufacturer’s instructions. RNA quantification was performed by NanoDrop spectrophotometer and reverse transcription in cDNA by “High-Capacity cDNA Reverse Transcription kit” (Thermo Fisher Scientific, Waltham, MA, USA, Cat# 4368813). BrightGreen qPCR MasterMix (Applied Biological Materials, Canada) was used for quantitative PCR assays, according to the manufacturer’s instructions on a CFX Connect Detection System (Bio-Rad, Hercules, CA, USA). Specific primer pairs were designed using the Oligo 4.0 software (Table 1) as already reported in Fasciolo et al., 2022 [22]. *Actb* and *B2m* were selected as housekeeping genes and the melt curves have been analyzed for assessing the specificity of all reactions. Relative gene expression variation was analyzed calculating ∆Ct (i.e., the difference between the mean Ct of reference genes and the Ct of testing gene) and applying the 2^−ΔΔCt^ method.

### 2.13. Data Analysis

All biochemical and blotting analyses are shown as means ± SEM. All experiments were performed in technical duplicate or triplicate and data analysed using the one-way analysis of variance (ANOVA) method and Tukey’s pairwise comparison tests. The differences with probability values (*p*) ≤ 0.05 were considered significant. All analyses were performed using GraphPad Prism 8.0.2 (GraphPad Software Inc., La Jolla, CA, USA).

For qPCR reactions, normal data distribution was assessed with the Shapiro–Wilk test (“shapiro.test function”, R language). The statistical significance of differences between control and testing samples or between two different groups (*p* value ≤ 0,05) was evaluated with a two-tailed (one sample and two samples, respectively) Student’s *t* test (GraphPad Software Inc., La Jolla, CA, USA). Differences were defined as significant at *p* value ≤ 0.05.

## 3. Results

### 3.1. Body Parameters

To test the efficacy of hormonal treatments, we first measured thyroid hormone levels (FT3 and FT4). Thyroid hormone administration significantly increased FT_3_ and decreased FT_4_ plasma levels, while Vit E did not affect them.

FT_3_ values were 4.05 ± 0.37, 3.41 ± 0.32, 26.10 ± 2.03 and 24.46 ± 2.18 pM, in C, C + VE, H and H + VE, respectively; as expected, exogenous administrated T_3_ exerted a negative feedback on the hypothalamic–pituitary–thyroid axis. In fact, FT_4_ values were 19.44 ± 0.51, 19.5 ± 0.51, 2.57 ± 0.13 and 2.44 ± 0.26 pM in C, C + VE, H and H + VE, respectively.

Vit E muscle content was increased both by thyroid hormone treatment and Vit E supplementation. In detail, the measured values were: 19.6 ± 0.65, 26.7 ± 1.45, 23.47 ± 0.50 and 31.9 ± 1.8 nmol/g tissue in C, C + VE, H and H + VE, respectively. The increased Vit E levels in H rats can be possibly due to the thyroid-hormone-induced mobilization of endogenous reserves or a higher assimilation of the vitamin from food [33].

### 3.2. Glucose Tolerance Test and Insulin Levels

To assess the onset of insulin resistance due to hyperthyroidism and the effect of Vit E treatments, we performed a glucose tolerance test on the different animal groups.

**Figure 1:**
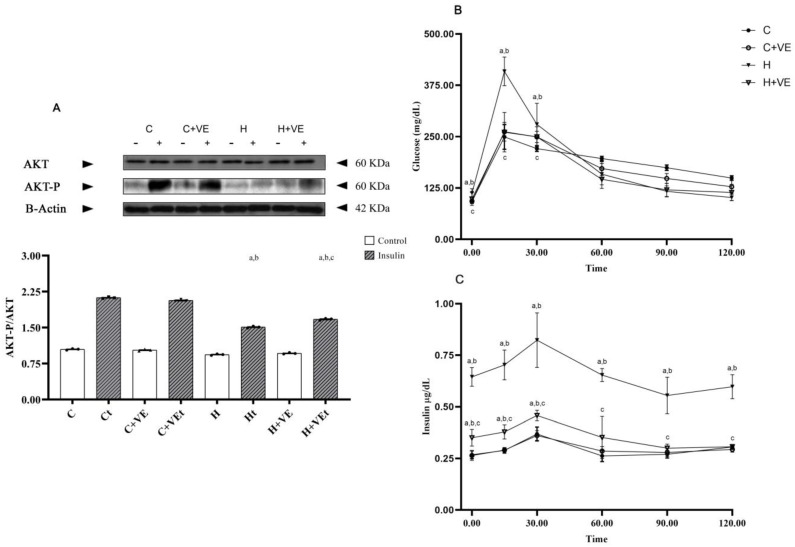
Panel (**A**): bottom phosphorylation degree of kinase AKT (AKT-P/AKT), in the absence and in the presence (I) of insulin; top, representative Western blot in the absence (−) and in the presence of insulin. Panel (**B**,**C**) plasma glucose and insulin during 120 min after intraperitoneal injection of glucose. C control rats; C + VE, vitamin-E-fed rats; H hyperthyroid rats; H + VE hyperthyroid and vitamin-E-fed rats. Values are the means ± SEM of six different rats in (**A**,**B**) and of three different rats in (**C**). *p* < 0.05 was chosen as the significance level. Meaning of the letters: a, *p* < 0.05 vs. C; b, *p* < 0.05 vs. C + VE; c. *p* < 0.05 vs. H (one-way ANOVA followed by Tukey post-test).

After 6 h of fasting, corresponding to the baseline (time 0) of the glucose tolerance test, the T_3_ treatment increased the basal blood glucose and plasma insulin level compared to C. Vit E administration to T_3_-treated animals shifted back the glucose levels to values comparable to C while also reducing the basal insulin level, although the treatment did not fully restore them to the basal condition (Figure 1B,C, time 0). Following glucose i.p. administration, the increase in the glucose plasma level (Figure 1B) was significantly higher in H in respect to C, C + VE and H + VE rats after 15 and 30 min.

The insulin plasma level (Figure 1C) was higher in H rats across all time points compared to the other groups. In H + VE, the increase in insulin level was blunted compared to that observed in H and remained higher than that observed in C and C + VE at time 0, 15 and 30 min.

These data indicate that the administration of thyroid hormones induces insulin resistance, and that Vit E limits this effect, despite not completely restoring insulinemia to the basal condition.

Moreover, to check if systemic insulin resistance could also be associated with skeletal muscle insulin resistance, we verified if in vitro exposure of muscle pieces to insulin was able to activate AKT (Figure 1A). No differences in AKT phosphorylation were observed among the groups in basal conditions. Indeed, insulin treatment increased AKT-P in all animal groups, despite its effect being significantly lower in H group vs. the C one. Notably, the administration of Vit E to hyperthyroid animals ameliorated skeletal muscle response to insulin, as evident by the increase in AKT-P/AKT in H + VE rats compared to H (Figure 1A). However, we noticed that the AKT-P/AKT ration in H + VE rats did not reach the ones observed in group C.

### 3.3. Oxidative Damage to Lipids and Proteins and Susceptibility to Oxidative Damage

**Figure 2:**
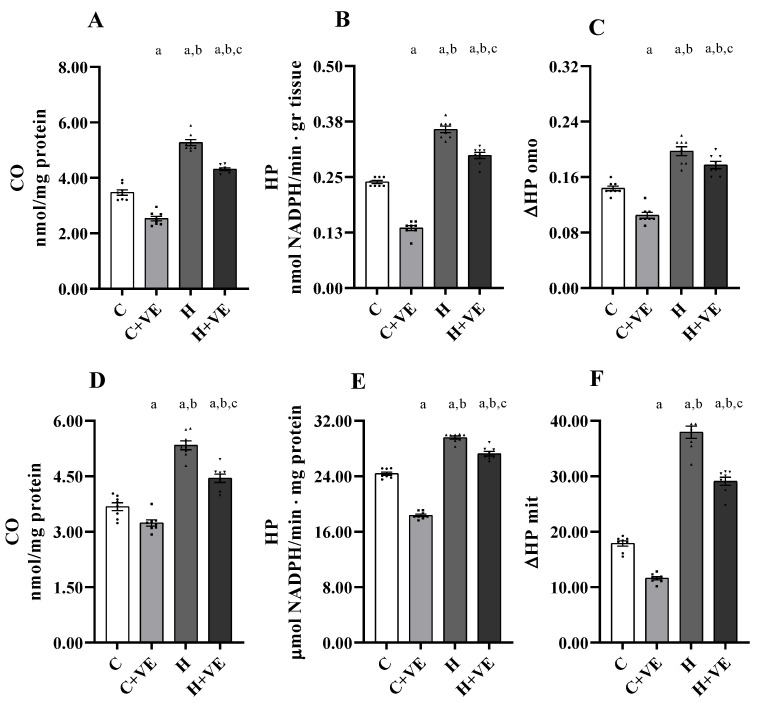
Oxidative stress markers (protein-bound carbonyls, CO, panels (**A**,**D**); and lipid hydroperoxides, HP, panels (**B**,**E**)) and in vitro response to oxidative stress (ΔHP, panels (**C**,**F**)), in skeletal muscle (**upper panels**) and mitochondria (**lower panels**). C control rats; C + VE, vitamin-E-fed rats; H hyperthyroid rats; H + VE hyperthyroid and vitamin-E-fed rats. Values are the means ± SEM of eight different rats. *p* < 0.05 was chosen as the significance level. Meaning of the letters: a, *p* < 0.05 vs. C; b, *p* < 0.05 vs. C + VE; c, *p* < 0.05 vs. H (one-way ANOVA followed by Tukey post-test).

The assessment of oxidative stress levels was performed by measuring the levels of lipids (hydroperoxides, HP; Figure 2B,E) and protein (protein-bound carbonyls, CO; Figure 2A,D) as markers of oxidative damage to lipids and protein. Since mitochondria are considered crucial actors of insulin resistance [34], we measured lipid and protein oxidation both in the tissue (Figure 2, top panels) and isolated mitochondria (Figure 2, bottom panels). Furthermore, we determined the in vitro susceptibility to oxidative stress by evaluating the changes in HP levels after an oxidative insult (ΔHP) in both muscle homogenates and mitochondria (Figure 2C,F, respectively).

Hyperthyroidism increased lipid and protein oxidative damage and the susceptibility to oxidative insult in vitro both in muscle homogenates and mitochondria. Interestingly, dietary Vit E supplementation reduced oxidative stress markers and susceptibility to oxidative stress when administered to both control and hyperthyroid rats.

### 3.4. Muscle Total ROS Content, Mitochondrial H_2_O_2_ Release and NADPH Oxidase (NOX) Activity

**Figure 3:**
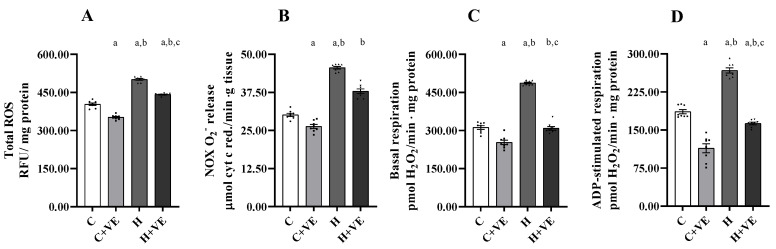
Total ROS content (**A**), NADPH oxidase (NOX) O^2−^ release (**B**), mitochondrial H_2_O_2_ release during basal (**C**) and ADP-stimulated respiration (**D**). C control rats; C + VE, vitamin-E-fed rats; H hyperthyroid rats; H + VE hyperthyroid and vitamin-E-fed rats. Values are the means ± SEM of eight different rats. *p* < 0.05 was chosen as the significance level. Meaning of the letters: a, *p* < 0.05 vs. C; b, *p* < 0.05 vs. C + VE; c, *p* < 0.05 vs. H (one-way ANOVA followed by Tukey post-test).

Increased oxidative stress in the skeletal muscle may depend on higher ROS production in the muscle, which, in turn, may be due to an enhanced production from the main cellular sources (NOX and mitochondria). Thus, we measured the total ROS content, the NOX activity and the mitochondrial H_2_O_2_ release (Figure 3).

The total ROS content (Figure 3A) was significantly increased by T_3_ treatment, whereas Vit E administration reduced it in both C and H rats.

T_3_ treatment also increased NOX activity, and Vit E supplementation (Figure 3B) reduced NOX activity in both control and hyperthyroid rats.

Mitochondrial ROS release was measured during basal (Figure 3C) and ADP-stimulated respiration (Figure 3D). The highest mitochondrial ROS release was observed in the H group, during basal and ADP-stimulated respiration. Vit E supplementation reduced ROS release during basal and ADP-stimulated respiration both when administered to C and H rats.

Overall, these data suggest that increased oxidative damage occurs in hyperthyroidism and that Vit E supplementation can rescue this damage at least in part by reducing ROS production.

### 3.5. Antioxidant Enzyme Activity of Skeletal Muscle Homogenate

**Figure 4:**
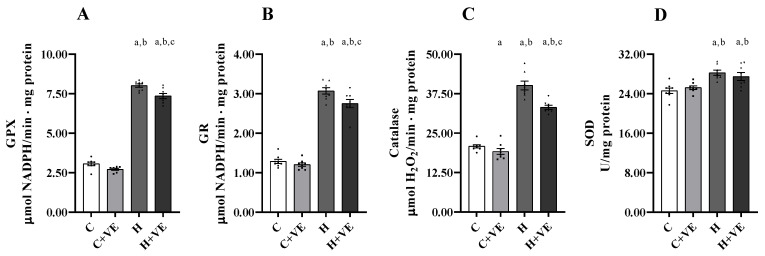
Antioxidant enzyme activities. GPX (panel (**A**)), GR (panel (**B**)) catalase (panel (**C**)), and SOD (panel (**D**)). C control rats; C + VE, vitamin-E-fed rats; H hyperthyroid rats; H + VE hyperthyroid and vitamin-E-fed rats. Values are the means ± SEM of eight different rats. *p* < 0.05 was chosen as the significance level. Meaning of the letters: a, *p* < 0.05 vs. C; b, *p* < 0.05 vs. C + VE; c, *p* < 0.05 vs. H (one-way ANOVA followed by Tukey post-test).

To assess the contribution of muscle antioxidant defense to the oxidative damage we measured the activities of the antioxidant enzymes glutathione peroxidase (GPX), glutathione reductase (GR), catalase and superoxide dismutase (SOD) (Figure 4).

The activities of all the antioxidant enzymes tested were increased in T_3_-treated animals. Vitamin E reduced the activity of the catalase in the C groups, and the activities of GPX, GR and catalase in H rats.

### 3.6. Oxygen Consumption of Skeletal Muscle Homogenate and Isolated Mitochondria

To assess the effects of treatments on oxygen consumption in the muscle homogenate and mitochondria, we evaluated oxygen consumption in the presence of pyruvate and malate as respiratory substrates in the absence (basal respiration) or in the presence of ADP (ADP-stimulated respiration). We also measured the respiratory control ratio (RCR), an index of the coupling between electron transport chain flow and ATP synthesis as well as mitochondrial integrity.

**Figure 5:**
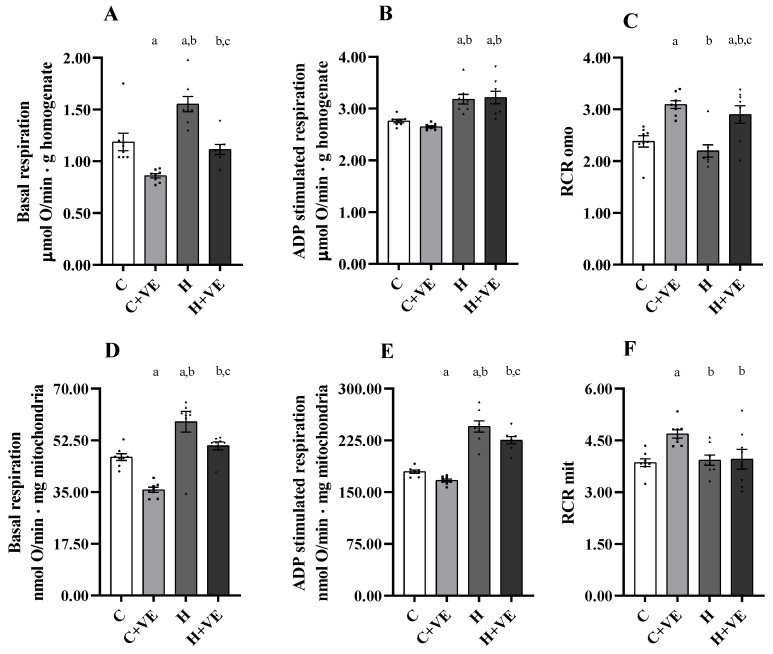
Basal and ADP-stimulated respiration and respiratory control ratio (RCR) detected in pyruvate plus malate supplemented homogenate (**A**–**C**) and mitochondria (**D**–**F**). C control rats; C + VE, vitamin-E-fed rats; H hyperthyroid rats; H + VE hyperthyroid and vitamin-E-fed rats. Values are the means ± SEM of eight different rats. *p* < 0.05 was chosen as the significance level. Meaning of the letters: a, *p* < 0.05 vs. C; b, *p* < 0.05 vs. C + VE; c. *p* < 0.05 vs. H (one-way ANOVA followed by Tukey post-test).

Thyroid hormone treatment increased homogenate and mitochondrial basal and ADP-stimulated respiration; the effect was observed in both H and H + VE groups vs. C (Figure 5A–E). Notably, Vit E significantly decreased homogenate basal respiration as can be deduced by comparing C + VE vs. C and H + VE vs. H (Figure 5A). No effect of Vit E on ADP-stimulated respiration was observed in homogenates (Figure 5B)

In the mitochondria, Vit E decreased the basal respiration rate in C + VE and H + VE rats (Figure 5D). Concerning ADP-stimulated, respiration a slight (8%) but significant reduction was observed (Figure 5E). Vit E increased the RCR in C + VE and H + VE homogenates (Figure 5C) and in the mitochondria of C + VE rats (Figure 5E). Thus, our data suggest that Vit E may counteract the increase in basal oxygen consumption due to thyroid hormone administration.

### 3.7. Factors Involved in Stress Response

**Figure 6:**
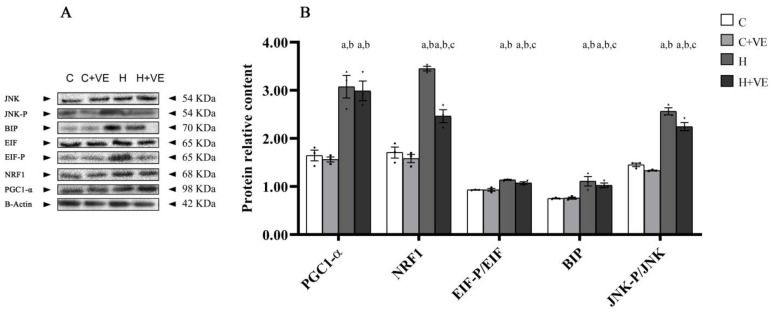
Oxidative stress sensitive factors involved in mitochondrial biogenesis (PGC1-α and NRF1) and cellular stress response (EIF, BIP JNK).A, representative Western blot images of PGC1-α, NRF1, EIF-P, EIF, BIP, JNK-P, JNK and β-Actin; B, muscle content of PGC1-α, NRF1, BIP and EIF-P/EIF and JNK-P/JNK ratio. C control rats; C + VE, vitamin-E-fed rats; H hyperthyroid rats; H + VE hyperthyroid and vitamin-E-fed rats. Values are the means ± SEM of three different rats. *p* < 0.05 was chosen as the significance level. Meaning of the letters: a, *p* < 0.05 vs. C; *b*, *p* < 0.05 vs. C + VE; c. *p* < 0.05 vs. H (one-way ANOVA followed by Tukey post-test).

To verify if oxidative stress affects putative factors involved in mitochondrial biogenesis and cellular stress response, we evaluated the changes in the levels of the peroxisome proliferator-activated receptor-gamma coactivator 1 alpha (PGC1-α), nuclear respiratory factor-1 (NRF1), BIP, and in the phosphorylation levels of the eukaryotic translation initiation factor 2 alpha (EIF) and c-Jun N-terminal kinase (JNK) (Figure 6B).

The phosphorylation level of EIF and JNK are reported as the ratio between the content of the phosphorylated and non-phosphorylated proteins (Figure 6B).

As reported in Figure 6, thyroid hormone treatment induced an increase in the content of PGC1-α, NRF1 and BIP, as well as in the phosphorylation of EIF and JNK. When administered to T_3_-treated rats (H + VE), Vit E reduced NRF1 and BIP content and the phosphorylation of EIF and JNK.

These data suggest a possible relationship between cellular stress and IR development in skeletal muscle.

### 3.8. Gene Expression Analysis of Slc2a1, Slc2a4, Pparg, Ppara, Cd36 and Il1b

**Figure 7:**
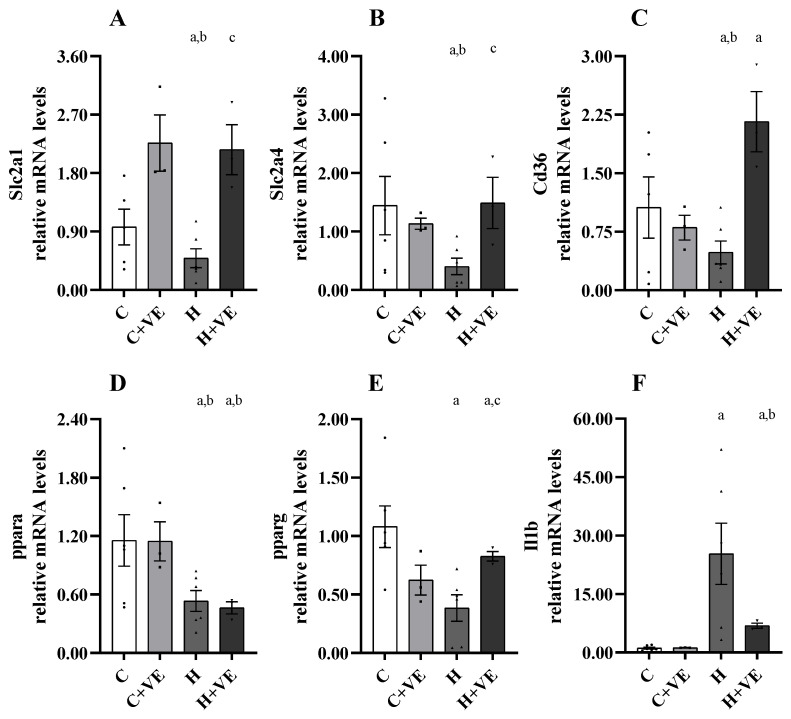
Relative mRNA quantification by qPCR of indicated genes (*Slc2a1*; *Slc2a4*; *Cd36*; *Ppara*; *Pparg*; *Il1b*, panels (**A**–**F**), respectively). C control rats; C + VE, vitamin-E-fed rats; H hyperthyroid rats; H + VE hyperthyroid and vitamin-E-fed rats. Expression values are shown as relative mRNA levels compared to C group applying 2^−ΔΔCt^ method. Actb and B2m were used as housekeeping genes. The histograms display for the different groups the means ± SEM. Normal data distribution has been assessed by Shapiro–Wilk test and the statistical significance (*p* value ≤ 0.05) was assessed by a two-tailed (one sample or two samples) Student’s *t* test (a, *p* < 0.05 vs. C; b, *p* < 0.05 vs. C + VE; c, *p* < 0.05 vs. H).

To identify putative metabolism-related genes perturbed by hyperthyroidism in the skeletal muscle and potentially modulated by Vit E, we analysed the expression of genes encoding glucose transporters (e.g., *Slc2a1* and *Slc2a4*), genes involved in muscle lipid homeostasis and insulin signalling (e.g., *Pparg, Ppara, Cd36*), as well as *Il1b,* known for its proinflammatory activity related to insulin response. As shown in Figure 7, the expression analysis revealed that all analyzed genes, except *Il1b* which was upregulated, were significantly repressed in muscle of H (vs. C) rats (Figure 7). Notably, we disclosed that Vit E in H rats efficiently induced the expression of muscular glucose transporters *Slc2a1* and *Slc2a4* (Figure 7), indicating, overall, the beneficial effects of Vit E supplementation on glucose uptake in the skeletal muscle of diseased animals. Moreover, in T_3_-treated rats, the Vit E administration also counteracted the strong upregulation of *Il1b* expression (Figure 7) and the reduction in *Pparg* (Figure 7).

## 4. Discussion

In the current work, we report that Vit E supplementation can attenuate hyperthyroidism-related changes in glycaemic and insulinemic response to a glucose load.

In our previous article, we suggested that antioxidant treatment can partially prevent the development of hepatic IR in hyperthyroid rats [22], and in this paper, we extended the concept to skeletal muscle.

Indeed, here, we observed that IR develops also in hyperthyroid muscle, as suggested by decreased AKT phosphorylation following insulin stimulation, in line with the observation that skeletal muscle plays a relevant role in the onset of whole-body IR [35] and support muscle involvement in hyperthyroidism-induced IR.

Hyperthyroidism-induced IR is also associated with increased oxidative damage in skeletal muscle, evident in the increase in lipid hydroperoxides and protein carbonyls content. The evidence that the antioxidant treatment, obtained by Vit E administration to hyperthyroid rats, reduces oxidative stress markers and muscular and systemic IR points toward the involvement of oxidative stress in IR development.

The enhancement of lipid peroxidation, observed in hyperthyroid rats, depends, at least in part, on the increase in fatty acid unsaturation degree [36] that makes them more prone to oxidation. Vit E reacts with lipid peroxyl radicals, thereby disrupting the propagation of lipid peroxidation chain reactions and contrasting the thyroid hormone effect.

Our data also shed light on the cellular sites responsible for the increased ROS content, observed in hyperthyroid skeletal muscle, that underlines the enhancement in lipid and protein oxidative stress. Indeed, we showed that in hyperthyroid animals, both an increase in NOX activity and in mitochondrial ROS release take place. Concerning NOX, the muscle fibres mainly express two isoforms of the enzyme: NOX2, mainly located at the plasma membrane and transverse tubules [37] and NOX4, mainly located at the sarcoplasmic reticulum, transverse tubules, and inner mitochondrial membrane [38]. NOXs have an important role in muscle function [39], but their increased activity may contribute to disease progression in pathologic conditions, such as IR. Indeed, in this context, it has been reported that the administration of the NOX inhibitor apocynin to streptomycin-induced diabetic rats reduces fasting blood glucose and enhances insulin sensitivity [40]. Thus, the increase in NOX activity observed in hyperthyroid animals may contribute to the occurrence of IR.

Our data also confirm that T_3_ treatment enhances mitochondrial ROS release [41]. This effect, plausibly, depends on changes in the mitochondrial biochemical composition induced by thyroid hormones, with an increase in the content of electron transporters, including those responsible for electron leakage and superoxide formation [42]. It has been suggested that at least three different pathways of target gene expression regulation contribute to thyroid hormone induced synthesis of mitochondrial components and mitochondrial biogenesis [43]. The first pathway starts upon T_3_ binding to nuclear receptors α and β (TRα1, TRα2 and TRβ) and consists of modulating gene transcription [44]. Upon T_3_ binding, the TRs act as homo- or heterodimers (when associated with retinoic acid receptors), bind to specific sites in the regulatory regions of target genes, named thyroid response elements (TRE), and recruit chromatin remodelling complexes, which modify histones and lead to interchange between the open (transcriptionally active) and closed (transcriptionally silenced) chromatin state [44]. In the skeletal muscle the TRα1 isoform is involved in the thyroid-hormone-induced induction of mitochondrial biogenesis [45]. In the second pathway, T_3_ has a direct effect on the mitochondrion via the binding to a mitochondrial-localised receptor. This receptor is an alternative translation product of the TRα gene (p43) and its overexpression increase mitochondrial biogenesis and protein synthesis [43]. The third pathway involves intermediate factors that are synthesized via thyroid TRE, enter the nucleus, and regulate a second series of thyroid hormone target genes. Among such intermediate factors are the transcription factors nuclear respiratory factor 1 and 2 (NRF1, NRF2) and the coactivators PGC-1α. These regulation mechanisms are additionally modulated by many non-genomic actions, e.g., post-translational modifications, or direct binding of thyroid hormone or its derivative to target structures [43].

The ability of Vit E to reduce ROS tissue levels is due to its capacity to reduce NOX activity and mitochondrial H_2_O_2_ release. The first effect may be due to the ability of Vit E to reduce the plasma membrane translocation of p47phox, the cytosolic component of the enzyme [46,47]. Concerning the Vit-E-induced reduction in mitochondrial H_2_O_2_ release, it may be due to the ability of the benzene ring to influence mitochondrial O_2_ and H_2_O_2_ generation by preventing electron loss and, thus, regulating superoxide production and/or by removing superoxide once formed. [48]. These actions reduce the likelihood of ROS formation and the triggering of peroxidation chain reactions [33].

In agreement with our previous data [49], we also report the increased activity of antioxidant enzymes (GPX, GR, Catalase and SOD) in hyperthyroid rats, that, however, is not sufficient to contrast the occurrence of oxidative damage.

In general, it appears that the activity of antioxidant enzymes is related to the extent of oxidative stress. In line with this, our data show that the reduction in oxidative stress markers induced by Vit E is accompanied by a reduction in the antioxidant enzyme activity. A similar relationship has been found in the ageing brain, where the increase in lipid peroxidation is accompanied by an increase in the activities of antioxidant enzymes [50].

These observations are in line with the evidence that, despite the increased activity of antioxidant enzymes, the susceptibility of skeletal muscle to oxidative insults remains higher in hyperthyroid rats, and that the administration of Vit E slightly reduces it. Therefore, the hyperthyroid skeletal muscle is more prone to damage and, possibly, to functional alterations.

It has been proposed that mitochondrial dysfunction and endoplasmic reticulum (ER) stress are key mechanisms in the emergence of IR in skeletal muscle [51]. Mitochondria are the primary target of the ROS they self-produce. Accordingly, our data show that the increased hyperthyroidism-induced mitochondrial ROS release results in higher oxidative damage to mitochondrial lipids and proteins, associated with increased susceptibility to stress. Despite the increase in signs of oxidative stress and the occurrence of IR in hyperthyroid rats, ADP-stimulated respiration increases in according to the well-known metabolic-stimulating effects of thyroid hormone.

Mitochondrial respiration rate, when detected in the whole homogenate, depends on both specific mitochondrial functionality and tissue mitochondrial content.

The administration of Vit E to hyperthyroid rats slightly reduced ADP-stimulated respiration when it was detected in isolated mitochondria but not when detected in whole tissue homogenates. This discrepancy could be more apparent than real since in our study we used mitochondria obtained at 3000× *g*, such fraction represents the most functional mitochondria that at the same time are the most ROS producer [52]. Thus, it is plausible that the slight effect of Vit E on ADP-stimulated respiration could be brought to light only when the measurement was performed on 3000× *g*-isolated mitochondria fraction, since all mitochondria populations contribute to respiration detected in the whole homogenate. Another possibility explaining the above-cited discrepancies is that Vit E, by reducing ROS, could interfere with a ROS-sensitive factor involved in mitochondrial biogenesis. In the whole our results appear in line with the observation that an increase in mitochondrial oxidants, independent of mitochondrial dysfunction, is sufficient to induce IR [53].

Mitochondrial oxygen consumption increases during basal respiration in the muscle of hyperthyroid rats and is reduced by antioxidant treatment. These changes may depend on variations in the inner mitochondrial membrane proton conductance, which are partly dependent on lipid peroxidation extent [54].

Increased ROS production and protein carbonylation can affect protein unfolding and upregulate the protein unfolding response [55]. Mitochondria and the endoplasmic reticulum are structurally and functionally connected [56,57,58,59] and this close interconnection implies that ROS generated in mitochondria promote ER stress [60].

GRP78/BiP is an important ER chaperone protein critical for ER protein quality control [61]. During oxidative stress, an integrated stress response (ISR), a stress-adaptive pathway, is activated, with EIF2α representing a crucial node [55]. EIF2α is activated by phosphorylation and plays a relevant role in mediating translation attenuation during mitochondrial dysfunction and ER stress aimed at reducing protein influx into the two organelles [55,62].

Increased EIF2α activation associated with increased protein levels of the ER stress marker GRP78 BIP is found in hyperthyroid muscle and suggests that increased IR correlates with the onset of ER stress. Indeed, Vit E supplementation reduces both ER and ISR stress and IR, confirming the important role of oxidative stress in the development of IR.

Furthermore, increased ROS production affects transcription of NRF2 target genes, including respiratory NRF1, which regulates mitochondrial gene expression [63,64]. The activity of these transcription factors is coordinated by PGC1-α coactivator, a member of the PGC1 coactivator family [65].

It has also been suggested that NRF1 could be directly activated by redox signalling [61]. Accordingly, we found that the NRF1 content increases in hyperthyroid animals and is attenuated by antioxidant supplementation.

We also measured the phosphorylation level of JNK, one of the most studied factors in IR obesity models [66]. The inhibition and total or partial deletion of JNK may reduce the occurrence of IR in non-alcoholic fatty liver disease induced by a high-fat diet [67]. Indeed, activated JNK phosphorylates the insulin receptor [68] and the insulin receptor substrate (IRS) [69,70] on serine and threonine residues. In this way, it inhibits the physiological pathway of insulin through steric hindrance. We found that in hyperthyroid rats, JNK phosphorylation is significantly increased compared to control animals and is attenuated by antioxidant supplementation.

Thus, our results agree with the observation that endogenous ROS act as potent inducers of JNK [71], confirming the correlation between increased oxidative stress and JNK phosphorylation.

To define if, and to what extent, Vit E perturbs the expression levels of crucial genes involved in metabolic processes/pathways relevant for skeletal muscle functionality in hyperthyroid rats, we focused on specific markers of glucose transport (e.g., *Slc2a1* and *Slc2a4*), glucose/lipid homeostasis, insulin signalling (e.g., *Ppara, Pparg,* and *Cd36*) and pro-inflammatory response (e.g., *Il1b*). All the above-mentioned markers were detectable at reasonable levels for a bona fide analysis. In hyperthyroid muscle, all the genes studied were repressed except for *Il1b* which was upregulated, in line with the reduced insulin sensitivity of T_3_-induced hyperthyroid rats [22]. We also disclosed that Vit E in hyperthyroid rats efficiently induced the expression of muscular *Slc2a1* and *Slc2a4*, overall suggesting the beneficial effects of Vit E supplementation on glucose uptake in the skeletal muscle of diseased animals. Moreover, in T_3_-treated rats, the Vit E administration also counteracted the strong upregulation of *Il1b* expression and the reduction in *Pparg*. In line with the anti-inflammatory activity of the transcription factor Pparγ and its capacity to induce multiple genes involved in glucose and lipid uptake/transport [72,73], the Vit-E-mediated induction of *Pparg* in hyperthyroid rats is mirrored by the increase in its target lipid transporter Cd36, and also may contribute, at least in part, to the Vit-E-mediated induction of glucose transporters in hyperthyroid rats. Finally, whereas we recently demonstrated the beneficial effect of Vit E supplementation on liver *Ppara* [22], we did not confirm this effect in the skeletal muscle, possibly due to the most relevant role of this nuclear receptor being in liver-associated processes [74].

## 5. Conclusions

In conclusion, we demonstrate that skeletal muscle plays a relevant role in IR in hyperthyroidism. Moreover, we suggest that oxidative stress is crucial for IR development in the hyperthyroid condition. Therefore, the thyroid-hormone-induced increase in metabolic capacities can predispose to cellular dysfunction including reduced insulin sensitivity in skeletal muscle. Antioxidant supplementation reducing oxidative stress also reduces the development of IR; therefore, the adequate antioxidant status may prevent IR development under oxidative stress conditions.

## Figures and Tables

**Table 1:** Specific primer pairs used for qPCR assays.

	Sense Primer (5′–3′)	Antisense Primer (5′–3′)
*1 Slc2a1*	GCGGGCTGTGCTGTGCTC	CCACAGCAACAGCAGCAG
*2 Slc2a4*	ACCAGACCCGCCCTTTGC	CTGAAGGGAGCCAAGCAC
*3 Cd36*	TTACTGGAGCCGTTATTGGTG	CCTTGATCTTGCTGCTATTCT
*4 Ppara*	CCACTTGAAGCAGATGACCT	CATTGCCAGGGGACTCATCT
*5 Pparg*	GTCGGATCCACAAAAAGAGTA	TTTGTCTGTTGTCTTTCCTGT
*6 Il1b*	AGGCTGACAGACCCCAAAAG	AAGCTCCACGGGCAAGACAT

## Data Availability

The data is available to anyone who requests it.
